# Supplementary material for: Cancer cell survival depends on collagen uptake into tumor-associated stroma
Source: Nat Commun. 2022 Nov 18;13:7078. doi: 10.1038/s41467-022-34643-5 (PMC9674701; doi:10.1038/s41467-022-34643-5)
Supplement: Supplementary file 1 — Supplementary Information [file 41467_2022_34643_MOESM1_ESM.pdf]

Supplementary Information

Cancer cell survival depends on collagen uptake into tumor-associated stroma

Kuo-Sheng Hsu[1], James M. Dunleavey[1], Christopher Szot[1], Liping Yang[1], Mary Beth Hilton[1,2], Karen Morris[1,2], Steven Seaman[1], Yang Feng[1], Emily M. Lutz[1], Robert Koogle[3], Francesco Tomassoni-Ardori[3], Saurabh Saha[4,9], Xiaoyan M. Zhang[4,10], Enrique Zudaire[1,11], Pradip Bajgain[1], Joshua Rose[5], Zhongyu Zhu[6,12], Dimiter S. Dimitrov[6,13], Frank Cuttitta[1], Nancy J. Emenaker[7], Lino Tessarollo[8], and Brad St. Croix[1,*]

[1]Tumor Angiogenesis Unit, Mouse Cancer Genetics Program (MCGP), National Cancer Institute (NCI), NIH, Frederick, MD 21702, USA.

[2]Basic Research Program, Leidos Biomedical Research Inc., Frederick National Laboratory for Cancer Research (FNLCR), Frederick, MD 21702, USA

[3]MCGP, NCI, Frederick, MD 21702, USA.

[4]BioMed Valley Discoveries, Inc, Kansas City, MO, 64111, USA

[5]Biomolecular Structure section, Center for Structural Biology, NCI, NIH, Frederick, MD, 21702 USA

[6]Protein Interactions Section, Cancer and Inflammation Program, NCI, NIH, Frederick, MD, 21702 USA

[7]Division of Cancer Prevention, NCI, NIH, Bethesda, MD, 20892, USA

[8]Neural Development Section, MCGP, NCI, NIH, Frederick, MD, 21702 USA

[9]Current address: Centessa Pharmaceuticals, Cambridge MA 02139, USA.

[10]Current address: Ikena Oncology, Cambridge, MA 02210, USA

[11]Current address: Janssen Pharmaceutical Companies, J&J, Spring House, PA, 19477, USA

[12]Current address: Lentigen Technology, Inc. 1201 Clopper Road, Gaithersburg, MD, 20878 USA

[13]Current address: Center for Antibody Therapeutics, University of Pittsburgh School of Medicine, Pittsburgh, PA 15261

[*]Correspondence:

Brad St. Croix, stcroixb@mail.nih.gov, Ph: 301-846-7456; Fax: 301-846-1290

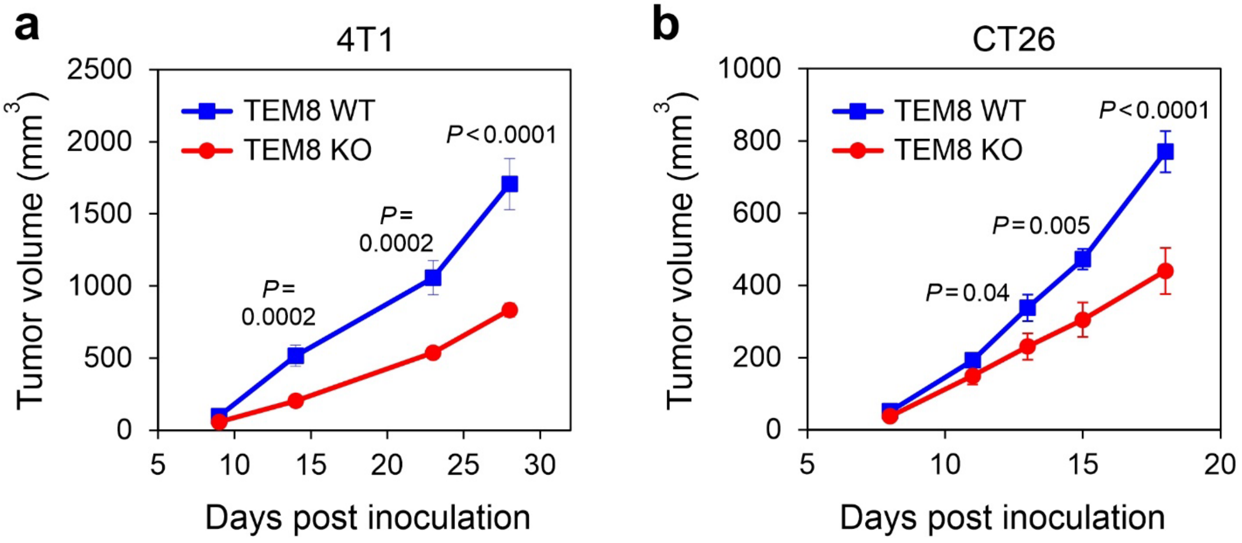

Supplementary Fig. 1 | TEM8 impacts tumor growth across various genetic backgrounds. **a**,**b**, Orthotopic growth of (**a**) 4T1 mammary or subcutaneous growth of (**b**) CT26 tumors in BALB/c TEM8 wildtype (WT) or knockout (KO) mice. Statistical analysis was calculated using unpaired *T* tests comparing tumor volume from TEM8 WT or KO mice at the same day post inoculation. n=14 (WT) or 16 (KO) (4T1), and 14 (WT) or 14 (KO) (CT26) biologically independent animals per group. Data are denoted as mean ± s.e.m. Source data are provided as a Source Data file.

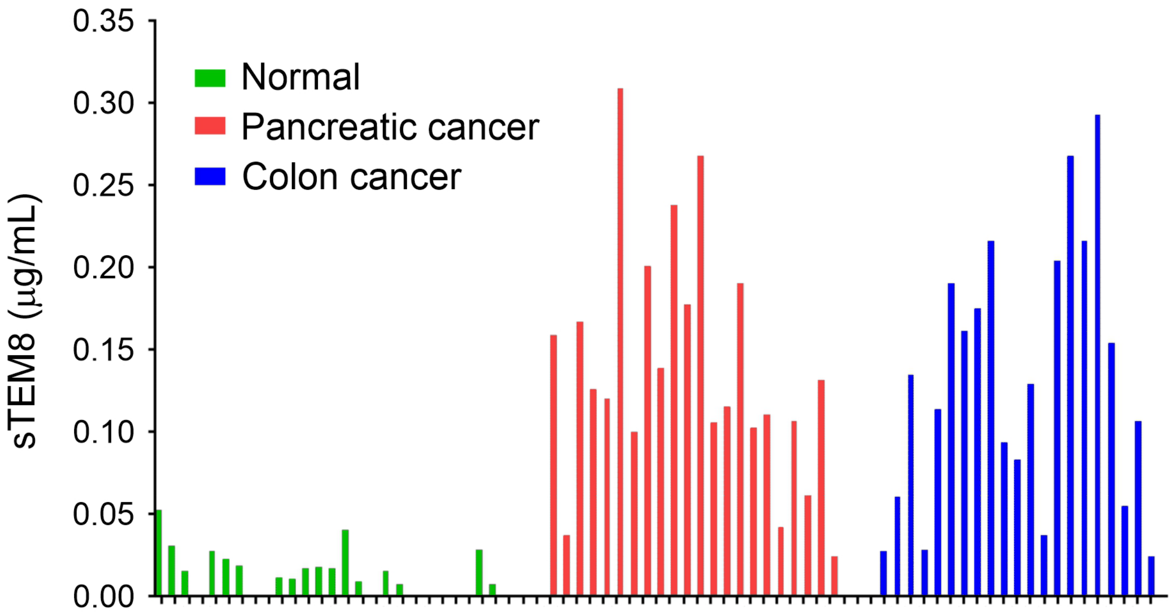

Supplementary Fig. 2 | An ELISA was used to detect soluble TEM8 (sTEM8) in serum from healthy controls (normal), or patients with pancreatic or colon cancer. N=26 (normal), 22 (colon cancer) and 21 (pancreatic cancer)/group. Soluble TEM8 was below detection in 9 of the normal samples.

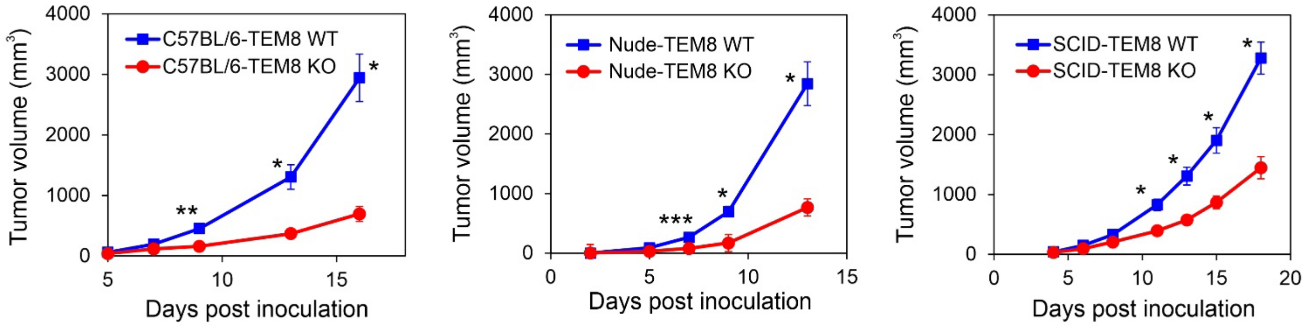

Supplementary Fig. 3 | Growth of MC38 tumors in TEM8 wildtype (WT) or knockout (KO) mice on a C57BL6, athymic nude, or SCID background. n=13 (B6, WT or KO), 16 (nu/nu, WT or KO), 13 (B6-SCID-WT) or 14 (B6-SCID-KO) biologically independent animals per group. *$P \leq 0.0001$, **$P < 0.0006$, ***$P < 0.0004$. Statistical analysis was calculated using unpaired *T* tests comparing tumor volume from TEM8 WT or KO mice at the same day post inoculation. Data are denoted as mean ± s.e.m. Source data are provided as a Source Data file.

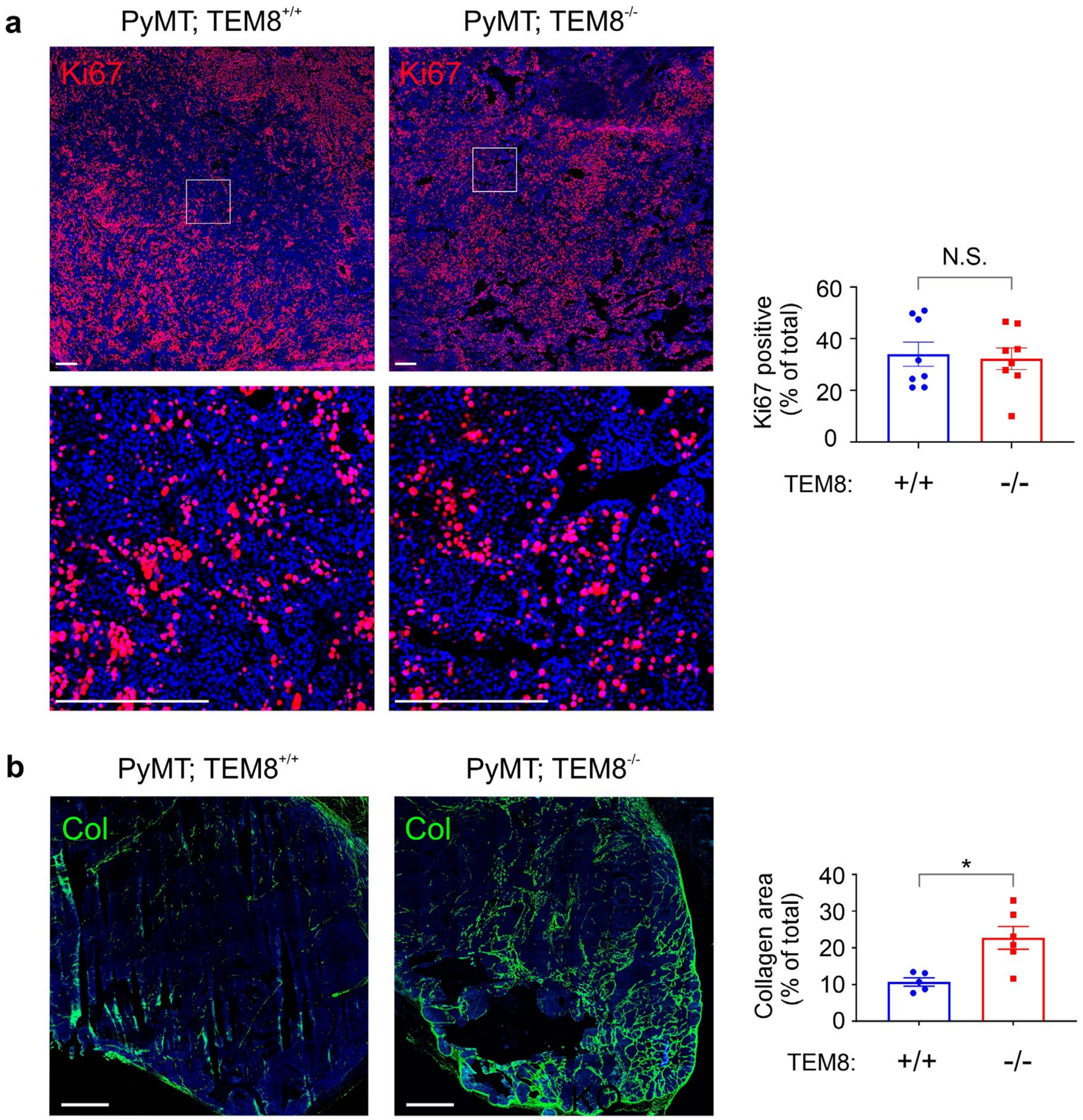

Supplementary Fig. 4 | Immunofluorescence staining of spontaneous MMTV-PyMT tumors. **a**, Immunofluorescence staining for Ki67 in orthotopic breast tumors from MMTV-PyMT transgenic TEM8 WT and KO mice. bar: 200 μm. Quantification is shown in the right panel. N.S.: non-significant. *n* = 8 independent samples per group. Statistical analysis was calculated by using an unpaired *T* test.
b, Immunofluorescence staining of col1 in orthotopic breast tumors from MMTV-PyMT transgenic TEM8 WT and KO mice. bar: 1mm Quantification is shown in the right panel. *p= 0.01, TEM8 +/+ versus -/-. *n* = 5 (+/+) or 6 (-/-) independent samples per group. Statistical analysis was calculated by using an unpaired *T* test.
Data in (**a**) and (**b**) are denoted as mean ± s.e.m.

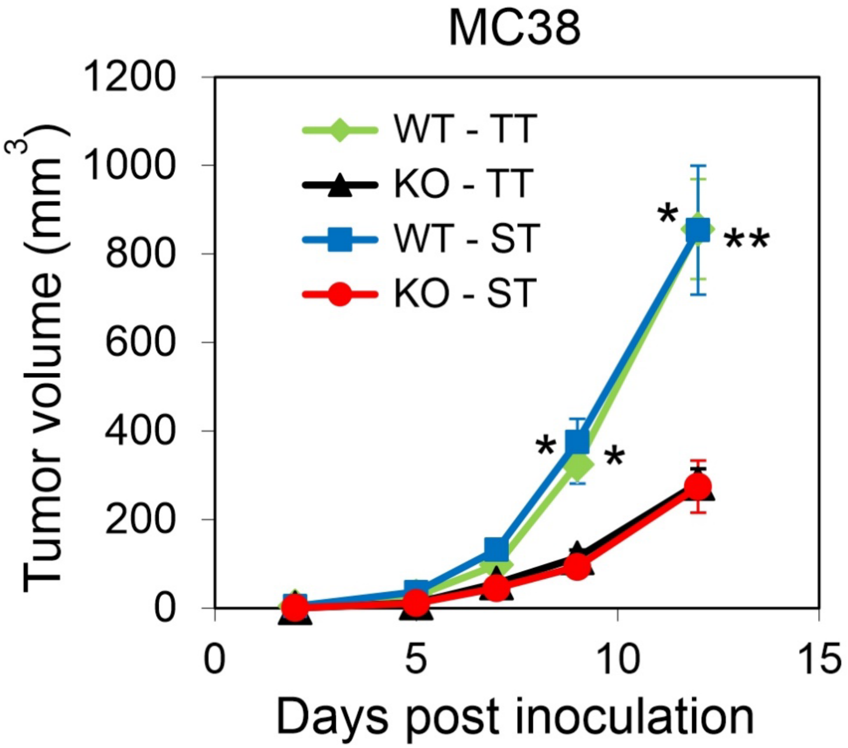

Supplementary Fig. 5 | Growth of MC38 tumors in C57BL6 TEM8 WT or KO mice housed at thermoneutral (22-23°C, TT) or subthermoneutral temperature (30-31°C ST). n=15/group *; $P<0.0001$ (KO vs WT comparison). **; $P=0.001$ (ST, KO vs WT comparison). p-values correspond to comparisons of tumor volumes between TEM8 WT and KO mice under TT or ST conditions at the same day post inoculation. Statistical analysis was calculated using unpaired *T* tests comparing tumor volume from TEM8 WT or KO mice at the same day post inoculation. No significant differences were observed between ST and TT mice. Data are denoted as mean ± s.e.m. Source data are provided as a Source Data file.

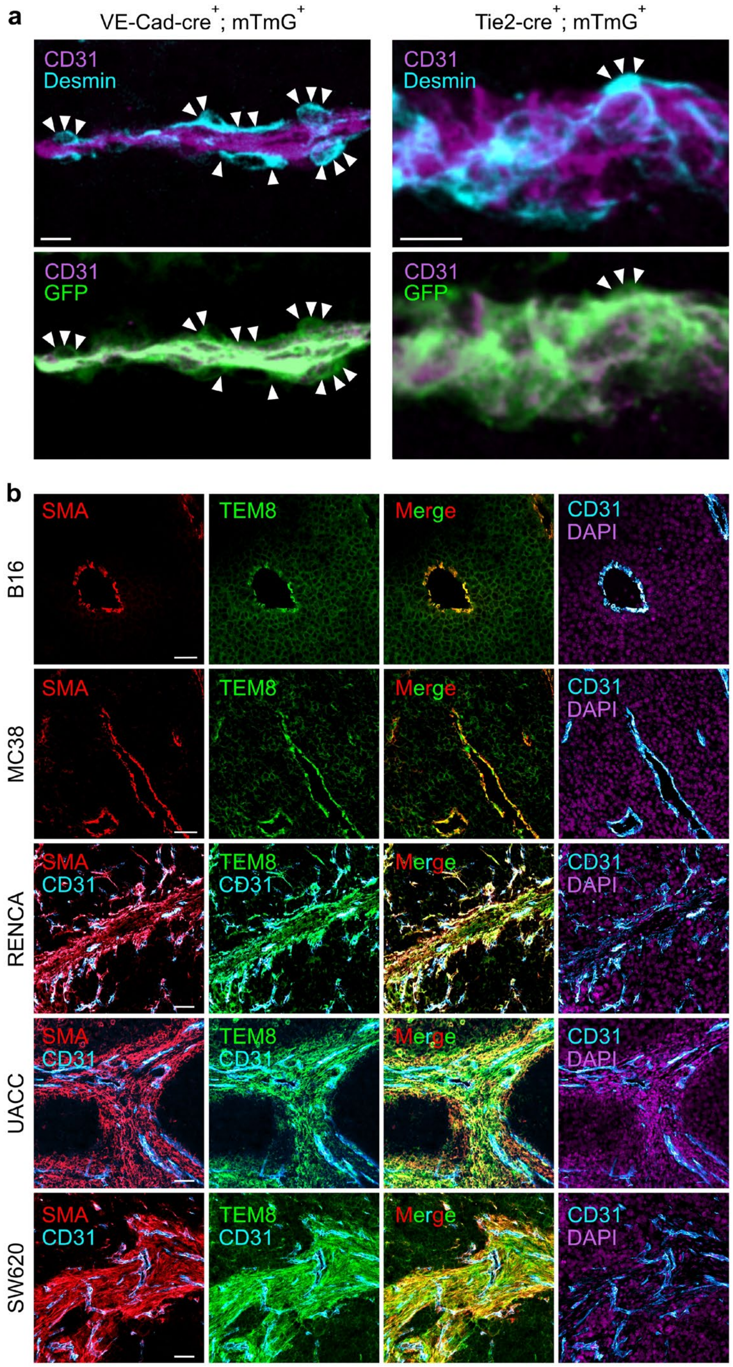

Supplementary Fig. 6 | CAF composition differs depending on the tumor model.
a, Immunofluorescence staining for GFP (green), an indicator of cre expression, in B16 tumors derived from mTmG reporter mice which also contain the VE-cad-cre (left panel) or Tie2-cre (right panel) transgene. Desmin (pseudocolored cyan) and CD31 (pseudocolored purple) were used to identify pericytes and endothelial cells respectively. The arrowheads highlight cre-positive pericytes. Bar: 10 μm.
b, Immunofluorescence staining for alpha smooth muscle actin (SMA) (red), TEM8 (green) CD31 (pseudocolored cyan) or DAPI (pseudocolored violet) in tumors used for conditional KO tumor studies. CAFs are defined as SMA^+ cells that reside outside vasculature. Bar: 50 μm. Images in (**a**) and (**b**) were representative of three experiments (*n* = 3 animals per group).

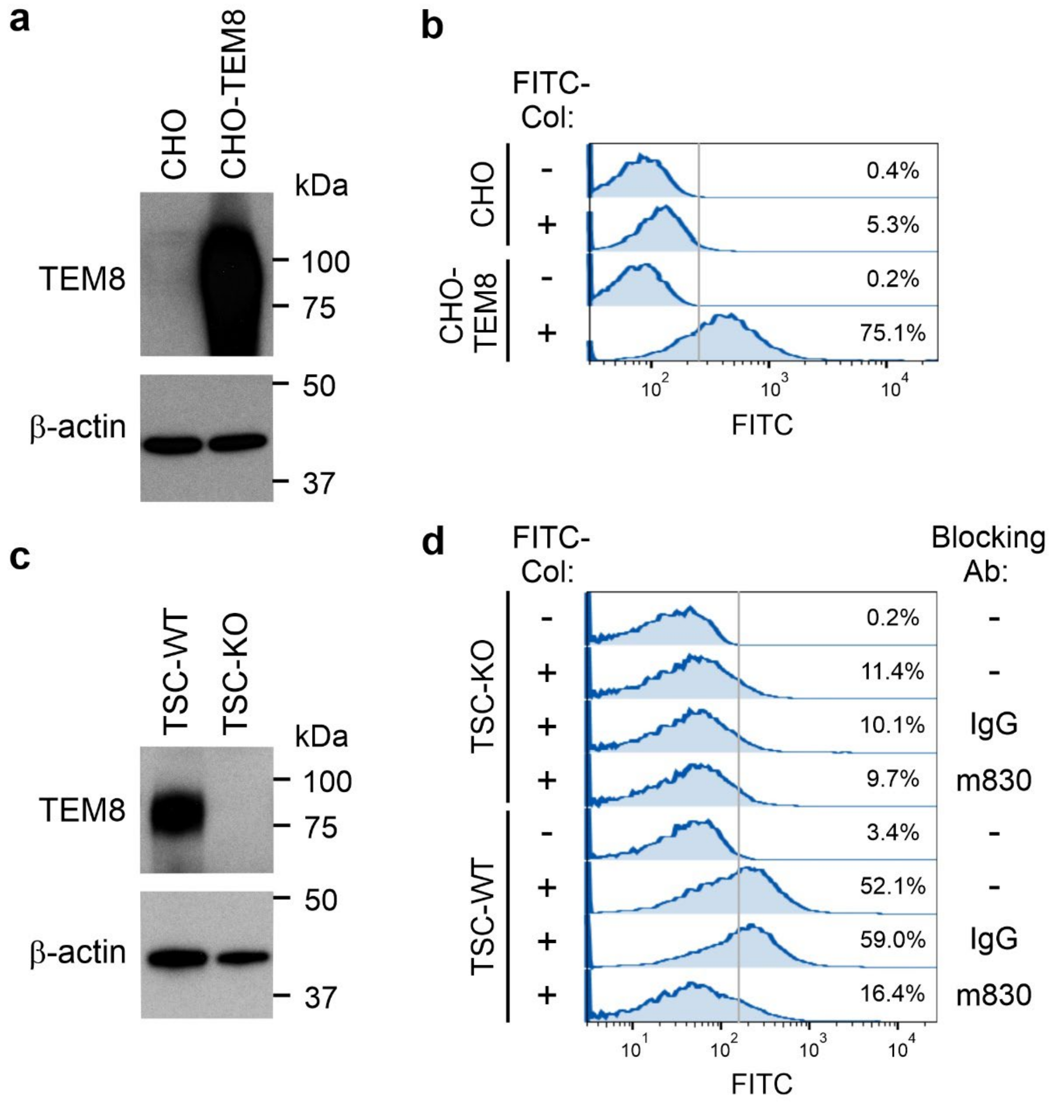

Supplementary Fig. 7 | TEM8 mediates collagen internalization into cells.
a, Western blotting was used to detect TEM8 expression in CHO and CHO-TEM8 cells. Images were representative of three experiments.
b, Uptake of FITC-Col into CHO and CHO-TEM8 cells was visualized using flow cytometry.
c, Western blotting was used to detect TEM8 expression in wildtype TSCs (TSC-WT) and TEM8 knockout TSCs (TSC-KO). Images were representative of three experiments.
d, Uptake of FITC-Col into TSC-WT and TSC-KO cells was visualized using flow cytometry.

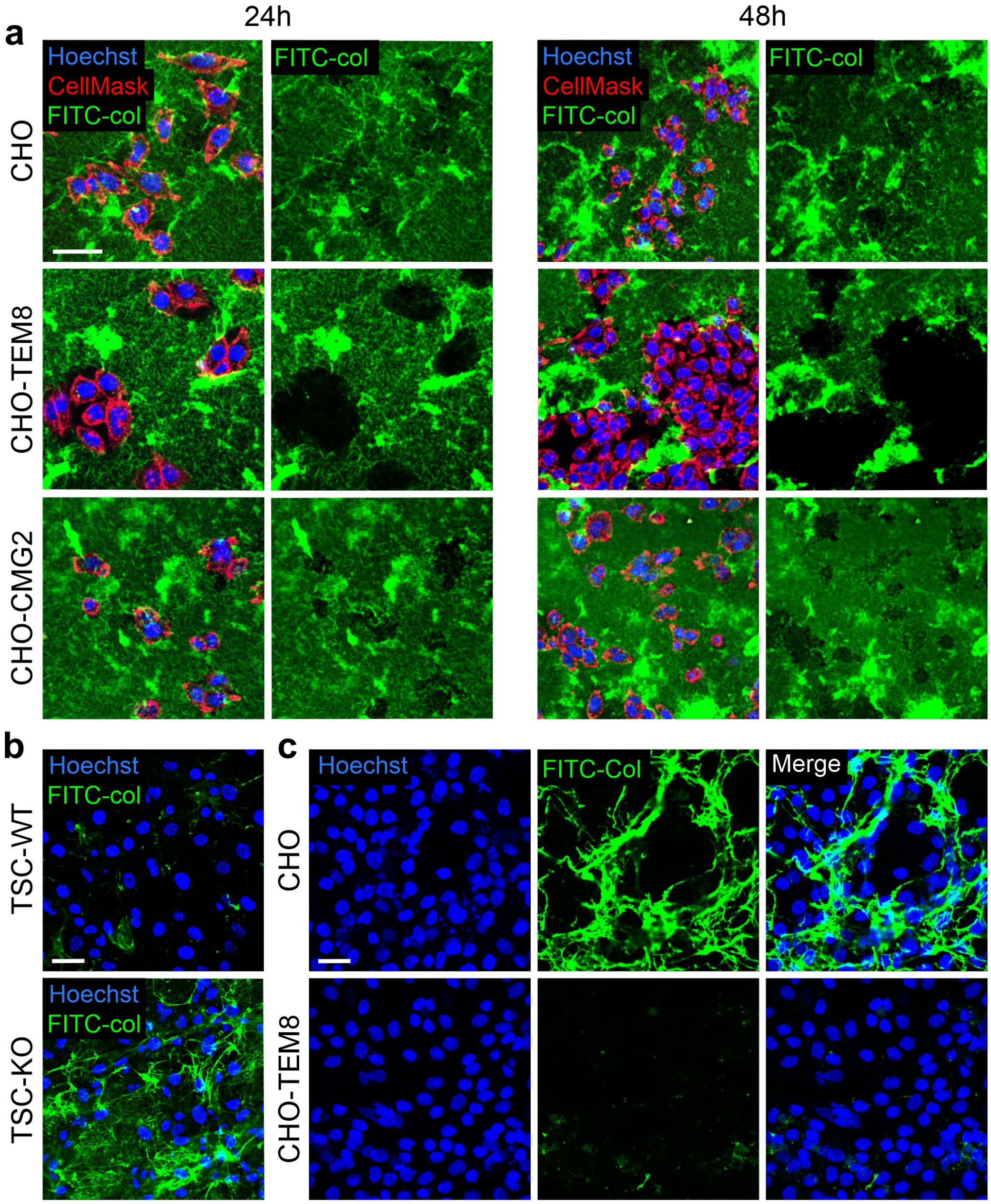

Supplementary Fig. 8 | TEM8 positive cells internalize and degrade FITC-collagen.
a, FITC-Col degradation by CHO, CHO-TEM8 and CHO-CMG2 cells was visualized by confocal microscopy after culturing cells on FITC-Col for 24 or 48h. Cells were stained with CellMask orange was used to visualize cell membranes (red) and Hoechst 33342 to visualize nuclei (blue). bar: 50μm.
b, FITC-Col degradation by TSC-WT or TSC-KO cells was visualized by confocal microscopy after culturing cells on FITC-Col for 24h. bar: 50μm.
c, FITC-Col was layered on top of CHO and CHO-TEM8 and degradation visualized 24h later by confocal microscopy. Cells were stained with DAPI to visualize nuclei (blue). bar: 50μm.
Images in (**a**) to (**c**) were representative of three experiments (*n* = 3 animals per group).

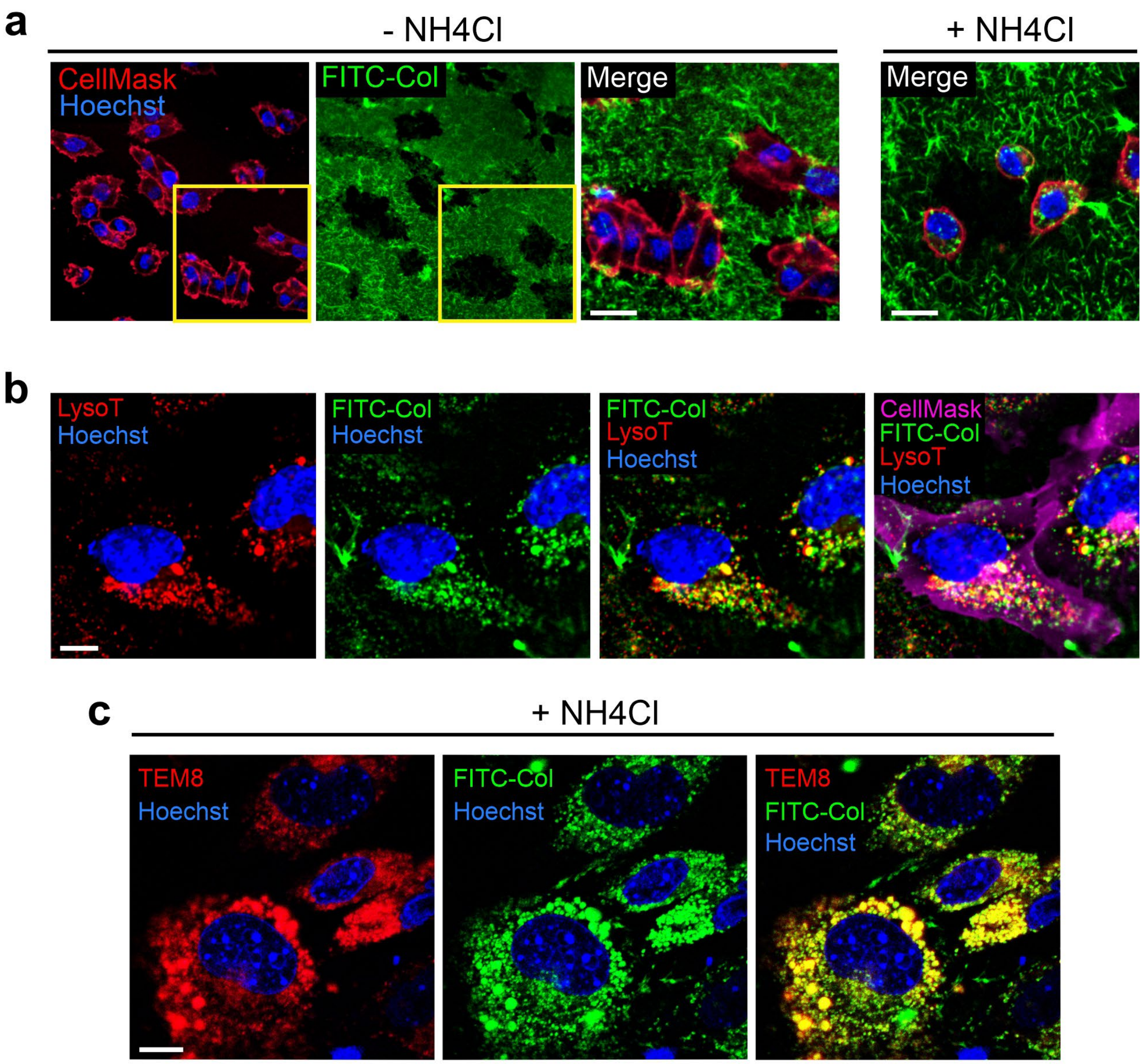

Supplementary Fig. 9 | Collagen 1 is internalized into lysosomes.
a, The ability of NH4CL to block FITC-Col degradation in lysosomes cells was assessed by confocal microscopy after culturing CHO-TEM8 cells on FITC-Col for 24h. Cells were stained with CellMask orange to visualize cell membranes (red) and DAPI to visualize nuclei (blue). bar: 50μm.
b, FITC-Col colocalization with LysoTracker Red (LysoT) was assessed by confocal microscopy after culturing TSC-WT cells on FITC-Col for 24h. Cells were stained with CellMask Red (pseudocolored violet) to visualize cell membranes and Hoechst 33342 to visualize nuclei (blue). bar: 50μm.
c, FITC-Col colocalization with TEM8 (red) was assessed by confocal microscopy after culturing TSC-WT cells on FITC-Col for 24h in the presence of NH_4Cl. Cells were stained with Hoechst 33342 to visualize nuclei (blue). bar: 50μm.
Images in (**a**) to (**c**) were representative of three experiments (*n* = 3 animals per group).

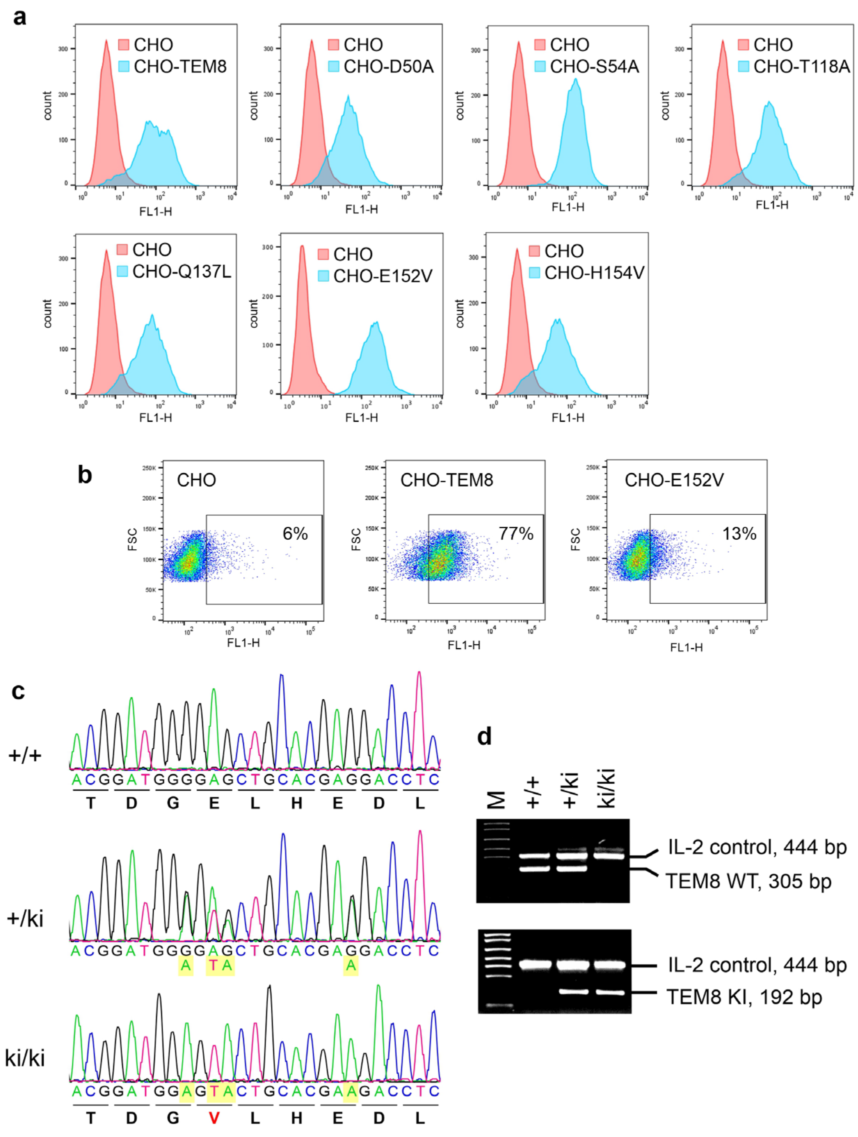

Supplementary Fig. 10 | The TEM8 E152V mutant is expressed on the cell surface and blocks collagen internalization.

a, Flow cytometry was used to measure surface levels of TEM8 in parental CHO cells (negative control) or CHO cell engineered to overexpression wildtype (WT) TEM8 or TEM8 mutant proteins.

b, Uptake of FITC-Col into CHO, CHO-TEM8 and CHO-E152V mutant cells was visualized using flow cytometry.

c, Chromatograms showing the sequence surrounding the E150V mutation in the germline of wildtype (+/+), heterozygous (+/ki) or homozygous (ki/ki) knock-in mice. Mutant nucleotides are highlighted in yellow, and the mutant valine (V) is highlighted in red.

d, A PCR assay was used to genotype the wildtype and knock-in alleles. Two PCRs were performed for each DNA sample, one for each allele, and an internal positive control (IL2) was included to confirm DNA integrity in samples without a test product.

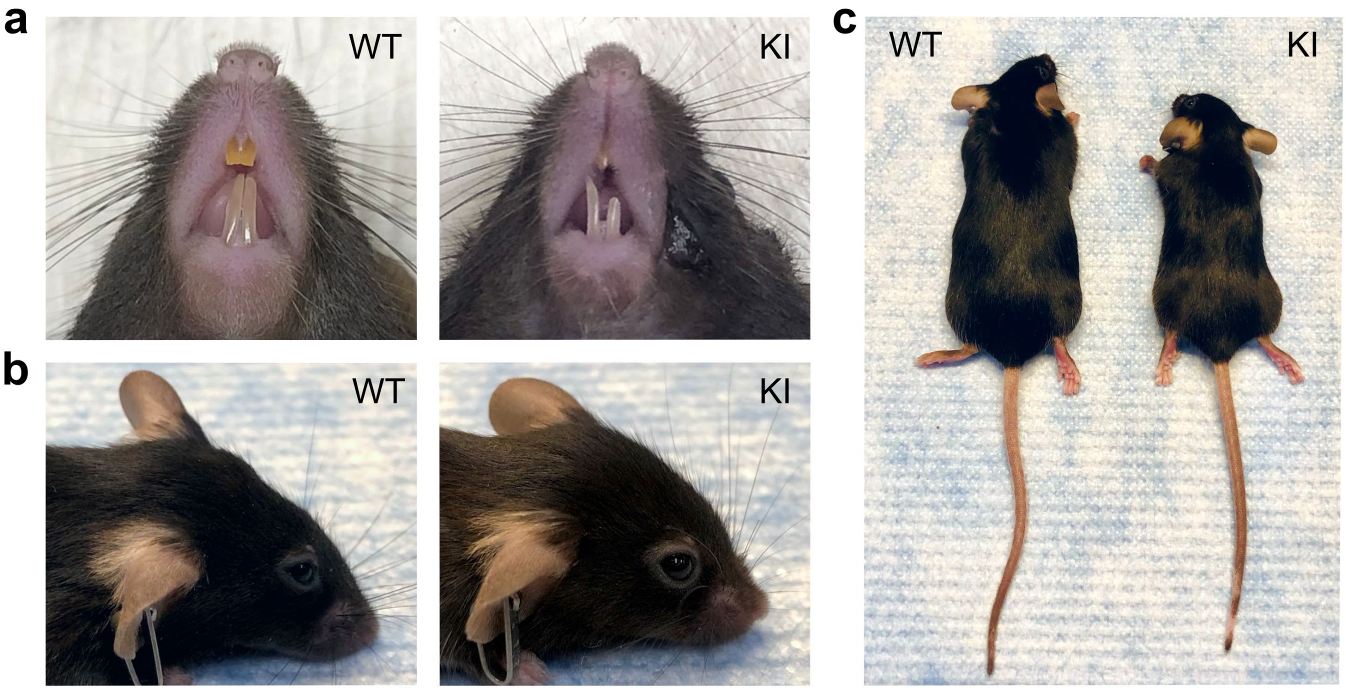

Supplementary Fig. 11 | E150V knockin mice display phenotypes found in TEM8 KO mice. **a**,**b**,**c**, TEM8$^{E150V/E150V}$ mutant knockin (KI) mice display (**a**) misaligned incisors (**b**), frontal bossing and (**c**) a reduced body size compared to TEM8 wildtype littermates.

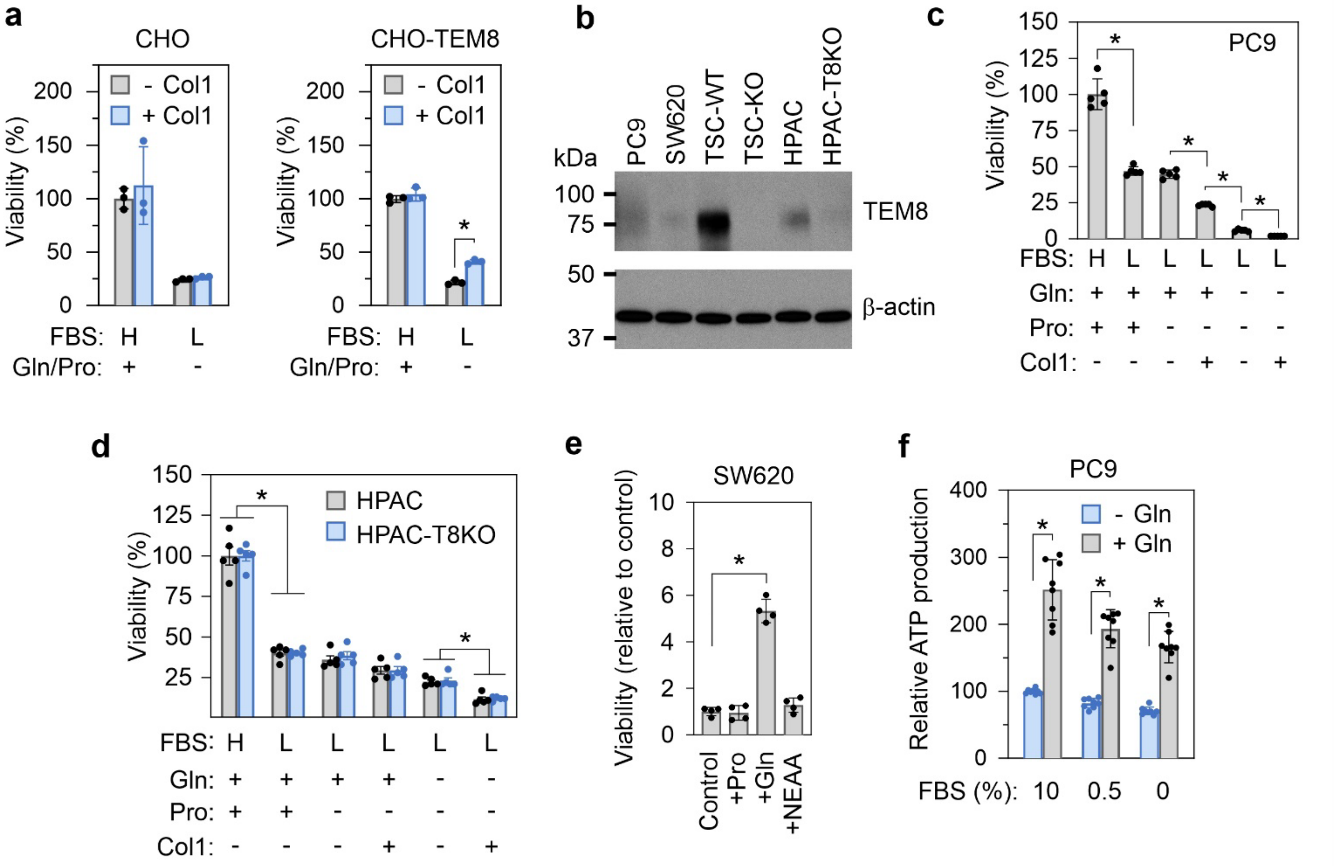

Supplementary Fig. 12 | Glutamine promotes the viability of nutrient starved cancer cells.
a, Viability of CHO and CHO-TEM8 cells grown in complete media or under nutrient starvation in the presence or absence of col1. *n* = 3 independent samples per group. Statistical analysis was calculated by using an unpaired *T* test. **P* = 0.0003.
b, Western blot was used to assess the relative levels of TEM8 in TSCs and tumor cell lines. β-actin was used as a loading control. Images were representative of three experiments.
c,**d**, Viability of PC9 (**c**) or HPAC and HPAC-TEM8 KO cells (**d**) under nutrient starvation in the presence or absence of col1. (**c**) and (**d**) **P*<0.0001. *n* = 5 independent samples per group. Statistical analysis was calculated by using one-way analysis of variance with a Tukey's test.
e, Viability of SW620 grown in basal DMEM with 0.5% FBS without (control) or with 2mM proline, 4mM Glutamine or a mixture of NEAAs (0.4 mM of Glycine, L-Alanine, L-Asparagine, L-Aspartic acid, L-Proline and L-Serine). The remaining 15 essential amino acids are present in the basal media. *p<0.0001. *n* = 5 independent samples per group. Statistical analysis was calculated by using one-way analysis of variance with a Tukey's test.
f, Relative ATP production in PC9 cells in the presence or absence of glutamine (Gln). *p<0.0001. *n* = 8 independent samples per group. Statistical analysis was calculated by using an unpaired *T* test.
Data are denoted as mean ± s.d. Source data are provided as a Source Data file.

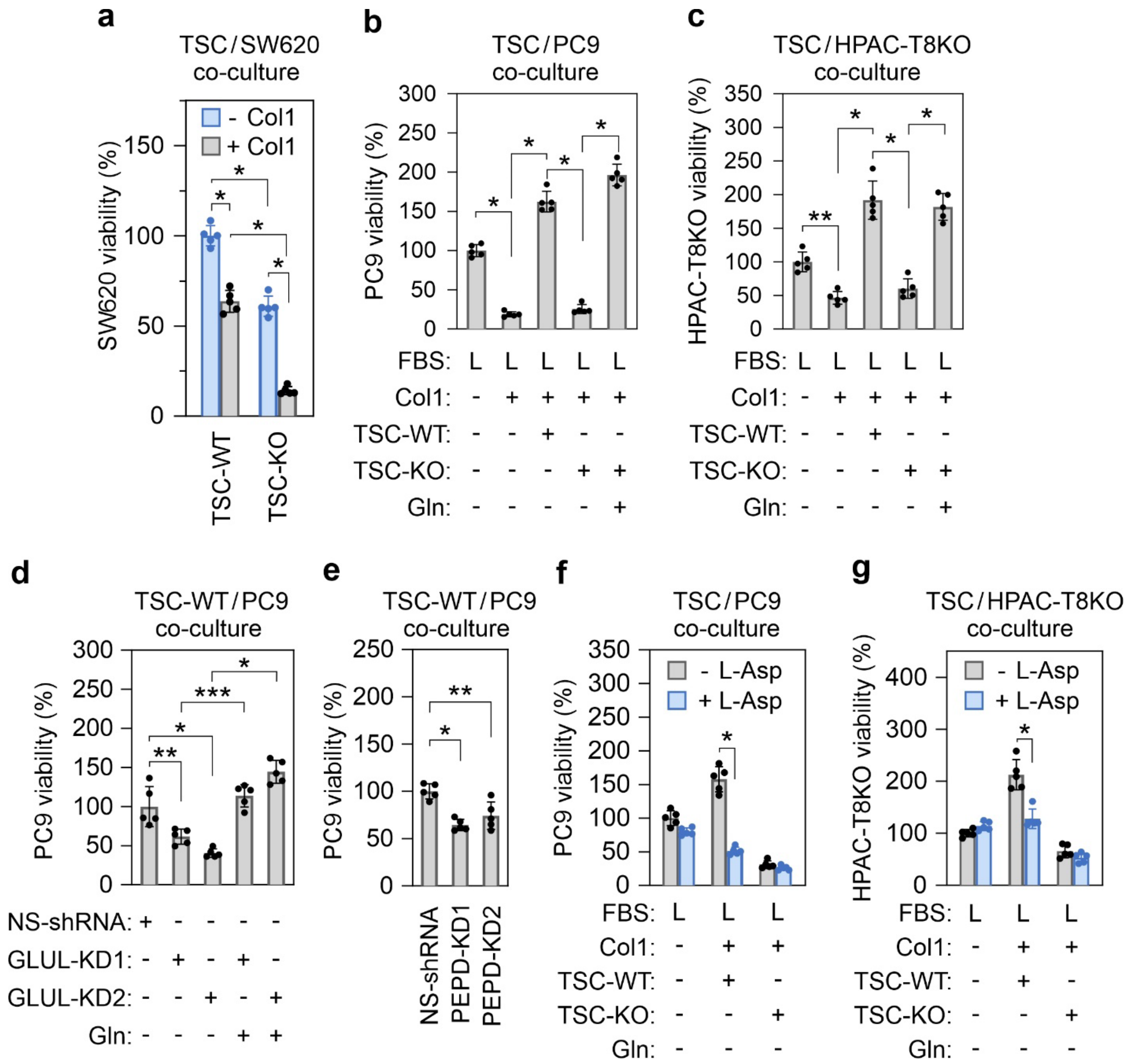

Supplementary Fig. 13 | TEM8⁺ TSCs promote the survival of nutrient starved cancer cells through the production of glutamine.

a, TEM8 WT TSCs help to protect nutrient starved SW620 cells from exogenous collagen toxicity. Cells were co-cultured in basal DMEM with 0.5% FBS with or without 40 μg/mL of exogenous collagen I. *n* = 5 independent samples per group. Statistical analysis was calculated by using one-way analysis of variance with a Tukey's test. **P*<0.0001

b,**c**, Viability of PC9 (**b**) or HPAC-TEM8 KO cells (**c**) cells under nutrient starvation in co-cultures with TEM8 wildtype or knockout TSCs. *n* = 5 independent samples per group. Statistical analysis was calculated by using one-way analysis of variance with a Tukey's test. **P* <0.0001, ***P* =0.0017.

d, Viability of PC9 under nutrient starvation upon co-culture with GLUL (**d**) knock down (KD) TSCs. NS-shRNA: non-specific control shRNA. *n* = 5 independent samples per group.

Statistical analysis was calculated by using one-way analysis of variance with a Tukey's test. **P* <0.0001, ***P* =0.0068, ****P* =0.0003.

e, Viability of PC9 under nutrient starvation upon co-culture with PEPD (**e**) knock down (KD) TSCs. NS-shRNA: non-specific control shRNA. *n* = 5 independent samples per group. Statistical analysis was calculated by using one-way analysis of variance with a Tukey's test. **P* <0.0004, ***P* =0.0048.

f,**g**, Viability of nutrient starved PC9 (**f**) or HPAC-TEM8 KO cells (**g**) in co-cultures with TSCs following treatment with L-Asparaginase (L-Asp). *n* = 5 independent samples per group. Statistical analysis was calculated by using one-way analysis of variance with a Tukey's test. **P* <0.0001.

Gln; glutamine, Pro; proline, H; high serum, 10% FBS, L; low serum 0.5% FBS.

Data are denoted as mean ± s.d. Source data are provided as a Source Data file.

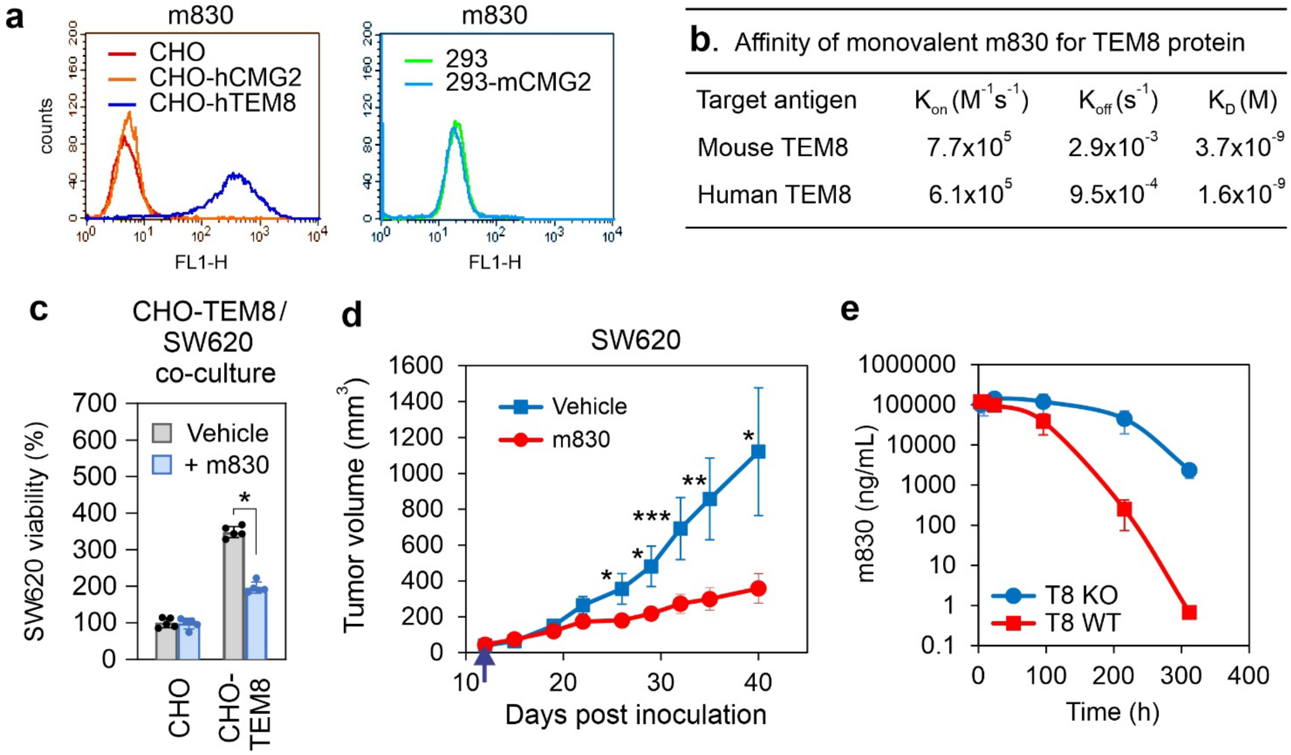

Supplementary Fig. 14 | TEM8-collagen neutralizing m830 antibody binds TEM8 and blocks tumor growth.

a, Flow cytometry was used to assess the cross-reactivity of m830 with mouse or human CMG2 using previously characterized cell lines[21].

b, Surface Plasmon Resonance (SPR) was used to calculate the binding affinity of m830 for mouse and human TEM8

c, The viability of SW620 cells in co-culture with CHO-TEM8 was measured in the presence or absence of 50 μg/mL of m830 antibody. In this experiment, cells were grown in 0.5% FBS, and in 0.5% FBS, and 0.5 mM glutamine. *n* = 5 independent samples per group. Statistical analysis was calculated by using an unpaired *T* test. *P<0.0001.

d, Growth of SW620 tumors following treatment with vehicle or m830 antibodies. Treatments (15 mg/kg 3x per week) began once tumors reached an average size of 40mm^3 (arrow). n=9 (vehicle) or 12 (m830) biologically independent animals per group. Statistical analysis was calculated by using an unpaired *T* test. *P=0.02, ** P= 0.01, ***P=0.03 between WT and KO at the indicated time point.

e, ELISA was used to detect m830 serum levels following intravenous injection of 5 mg/k into C57BL6 TEM8 KO versus WT mice. *n* = 3 to 5 independent animals per time point per group. Data in (**c**,**e**) denoted as mean ± s.d. Data in (**b**) denoted as mean ± s.e.m. Source data are provided as a Source Data file.

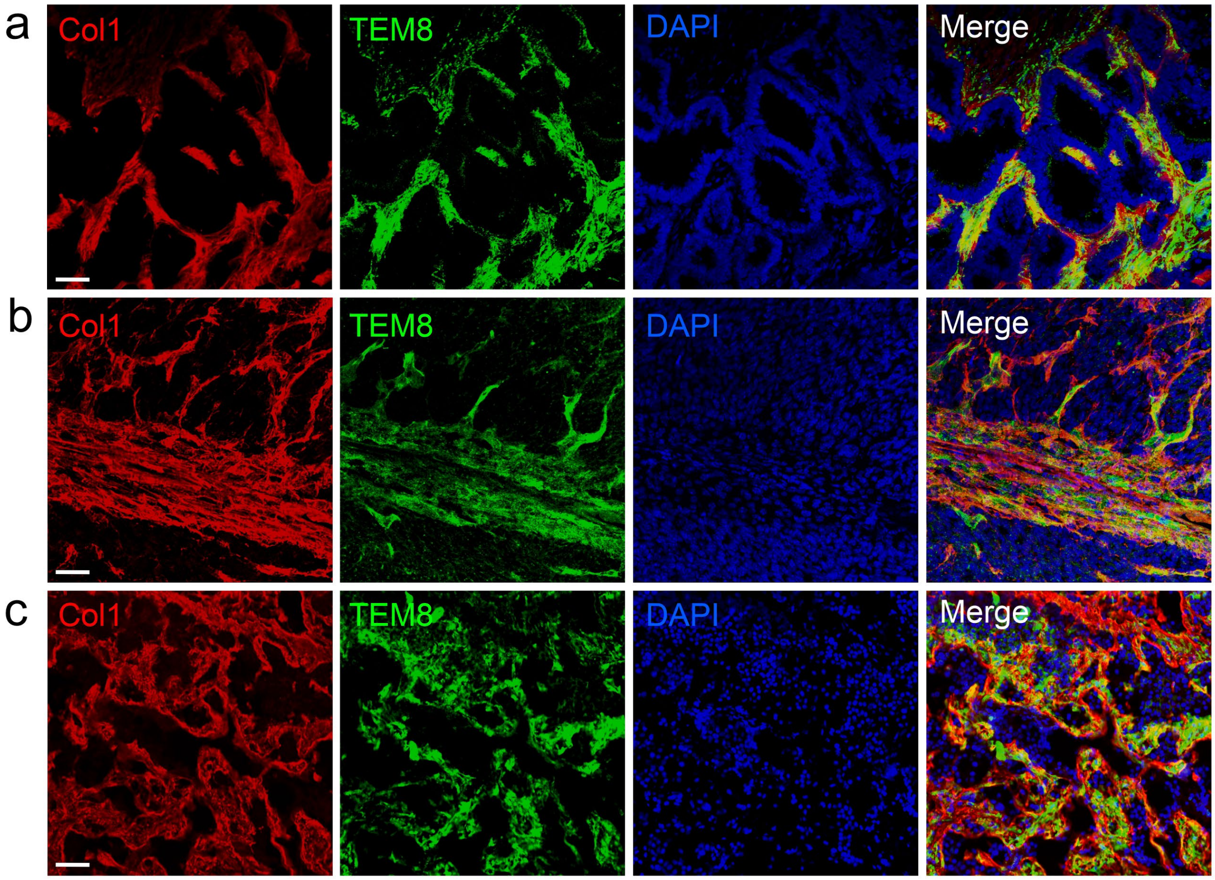

Supplementary Fig. 15 | TEM8 and collagen are highly expressed in colon cancer liver metastasis.
a,**b**,**c**, Immunofluorescence staining for Col1 (red), TEM8 (green) or nuclei (DAPI, blue) in (**a**) human colon cancer liver metastasis, (**b**) MC38 colon cancer liver metastasis in syngeneic mice or (**c**) HCT116 human colon cancer liver metastasis in athymic nude mice. bar: 50 μm. Images in (**a**) to (**c**) were representative of three experiments (*n* = 3 animals per group).

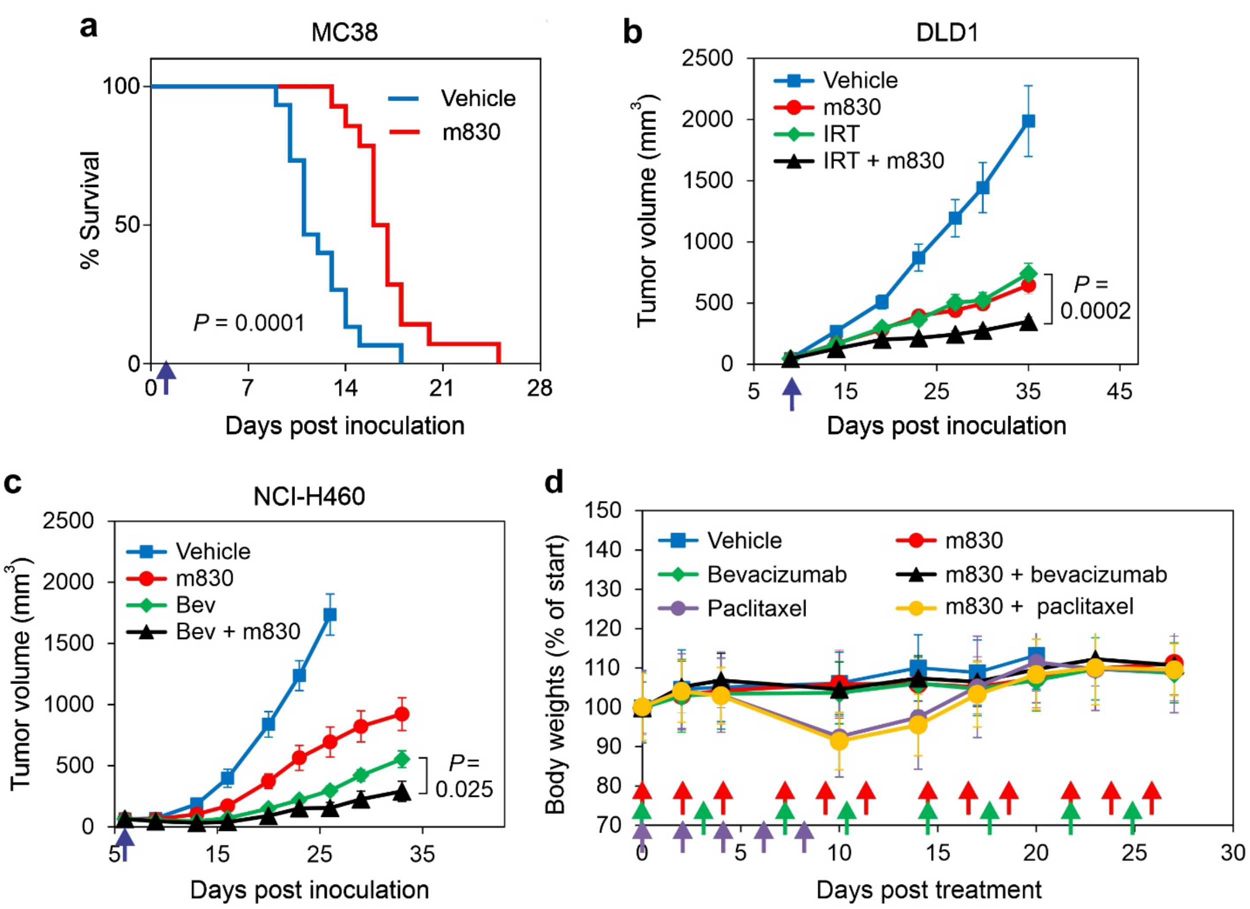

Supplementary Fig. 16 | TEM8-collagen neutralizing antibodies block tumor growth and metastasis without toxicities.

a, Kaplan-Meier survival analysis of mice with MC38 colon cancer liver metastasis. Treatments with 5 mg/kg of m830 began 1 day after intrasplenic tumor cell inoculation (arrow) and were administered 3x per week. Log-rank analysis: $P = 0.0001$ m830 versus vehicle. n=15 (vehicle) or 14 (m830) biologically independent animals per group.

b, Growth of DLD1 colon tumors following treatment with 15mg/kg of m830 3x per week, 80 mg/kg irinotecan (IRT) once per week, or a combination of m830 and IRT. Treatments were initiated (arrow) when tumors reached an average size of $50mm^3$. Statistical comparison of IRT vs. IRT + m830 was calculated at day 35 using an unpaired *T* test. n=18 (vehicle), 13 (m830), 14 (IRT) and 14 (IRT + m830) biologically independent animals per group.

c Growth of NCI-H460 lung tumors following treatment with 15mg/kg of m830 3x per week, 5 mg/kg bevacizumab (Bev) twice per week, or a combination of m830 and Bev. Treatments were initiated (arrow) when tumors reached an average size of $60mm^3$. Statistical comparison of Bev vs. Bev + m830 was calculated at day 33 using an unpaired *T* test. n=10 biologically independent animals per group.

The tumor studies in (**b**) and (**c**) were performed concurrently with those in **Fig. 7i,j** (main text) and the control and m830 groups were duplicated here for comparison.

d, Body weights from the NCI-H460 tumor study shown in (**c**) and **Fig. 6j** (main text) were monitored from the start of therapy until the tumors in the control groups reached their maximum allowable size and mice had to be euthanized.
Arrows indicate treatment days for m830 (red) bevacizumab (green) and paclitaxel (purple). While paclitaxel treatment alone resulted in a decreased body weight of ~10% versus vehicle at day 10 and 14 for, the addition of m830 to paclitaxel did not alter the level of weight loss.
Values in (**b** and **c**) denote mean ± s.e.m. Data in (**d**) denote mean ± s.d.
Source data are provided as a Source Data file.
