# Supplementary material for: Cancer cell survival depends on collagen uptake into tumor-associated stroma
Source: Nat Commun. 2022 Nov 18;13:7078. doi: 10.1038/s41467-022-34643-5 (PMC9674701; doi:10.1038/s41467-022-34643-5)
Supplement: Supplementary file 3 — Reporting Summary [file 41467_2022_34643_MOESM3_ESM.pdf]

nature portfolio

Corresponding author(s): Brad St. Croix
Last updated by author(s): 2022/10/4

Reporting Summary

Nature Portfolio wishes to improve the reproducibility of the work that we publish. This form provides structure for consistency and transparency in reporting. For further information on Nature Portfolio policies, see our Editorial Policies and the Editorial Policy Checklist.

Statistics

For all statistical analyses, confirm that the following items are present in the figure legend, table legend, main text, or Methods section.

n/a	Confirmed	
☐	☒	The exact sample size (*n*) for each experimental group/condition, given as a discrete number and unit of measurement
☐	☒	A statement on whether measurements were taken from distinct samples or whether the same sample was measured repeatedly
☐	☒	The statistical test(s) used AND whether they are one- or two-sided<br>*Only common tests should be described solely by name; describe more complex techniques in the Methods section.*
☒	☐	A description of all covariates tested
☒	☐	A description of any assumptions or corrections, such as tests of normality and adjustment for multiple comparisons
☐	☒	A full description of the statistical parameters including central tendency (e.g. means) or other basic estimates (e.g. regression coefficient) AND variation (e.g. standard deviation) or associated estimates of uncertainty (e.g. confidence intervals)
☐	☒	For null hypothesis testing, the test statistic (e.g. *F*, *t*, *r*) with confidence intervals, effect sizes, degrees of freedom and *P* value noted<br>*Give P values as exact values whenever suitable.*
☒	☐	For Bayesian analysis, information on the choice of priors and Markov chain Monte Carlo settings
☒	☐	For hierarchical and complex designs, identification of the appropriate level for tests and full reporting of outcomes
☒	☐	Estimates of effect sizes (e.g. Cohen's *d*, Pearson's *r*), indicating how they were calculated

Our web collection on statistics for biologists contains articles on many of the points above.

Software and code

Policy information about availability of computer code

Data collection	Flow cytometry data were acquired with BD Calibur and LSRII (BD).<br>Confocal images were collected with ZEISS LSM780.<br>Data for ELISA and viability assays were collected on a ClarioStar plate reader.<br>Bioluminescence Image of living animals was captured with IVIS Spectrum In Vivo Imaging System from Perkinelmer.<br>LC/MS/MS was performed with a Shimadzu 20AC-XR system (LC) and a TSQ Quantiva triple quadrupole mass spectrometer (Thermo Scientific)
Data analysis	Flow cytometry data were analyzed in FlowJo (v10).<br>ZEN 2.6 (Blue edition) was used to analyze confocal images.<br>Living Image® advanced in vivo imaging software from PerkinElmer (v 4.3.1.0.15880) was used for bioluminescence imageing of live animals.<br>Thermo Xcalibur Quan Browser software (version 4.3).<br>All statistical analysis was performed with GraphPad Prism 9.2.0.

For manuscripts utilizing custom algorithms or software that are central to the research but not yet described in published literature, software must be made available to editors and reviewers. We strongly encourage code deposition in a community repository (e.g. GitHub). See the Nature Portfolio guidelines for submitting code & software for further information.

Data

Policy information about availability of data

All manuscripts must include a data availability statement. This statement should provide the following information, where applicable:

- Accession codes, unique identifiers, or web links for publicly available datasets
- A description of any restrictions on data availability
- For clinical datasets or third party data, please ensure that the statement adheres to our policy

The data that support the findings of this study are available within the Article, Supplementary Information, or Source Data file. Source data are provided with this paper. The mouse strains generated in this study have been deposited to the Jackson Laboratory under Stock No. 037486 (B6-TEM8-flox), Stock No. 037488 (FVB-TEM8-flox), Stock No. 037490 (BALB/c-TEM8-flox) or Stock No. 037492 (B6-Tem8-E150V KI). All data reported in this paper will be shared by the lead author upon request. Some materials may require requests to collaborators and/or agreements with various entities. Materials that can be shared will be released via a Material Transfer Agreement.

Human research participants

Policy information about studies involving human research participants and Sex and Gender in Research.

Reporting on sex and gender	NA
Population characteristics	NA
Recruitment	NA
Ethics oversight	Anonymized human serum samples were obtained from the Research Donor Program (RDP) at the Frederick National Laboratory, the Cooperative Human Tissue Network (CHTN), or were a kind gift from Dr. Oliver Bathe (University of Calgary) with approval from the NIH Office of Human Subject Research (OHSR). Anonymized human FFPE normal or cancer tissue samples were obtained from the Cooperative Human Tissue Network (CHTN) with approval from the NIH OHSR. All clinical protocols were approved by institution-specific investigational review boards, with appropriate patient informed consent. Anonymized samples were provided without patient demographics.

Note that full information on the approval of the study protocol must also be provided in the manuscript.

Field-specific reporting

Please select the one below that is the best fit for your research. If you are not sure, read the appropriate sections before making your selection.

☒ Life sciences ☐ Behavioural & social sciences ☐ Ecological, evolutionary & environmental sciences

For a reference copy of the document with all sections, see nature.com/documents/nr-reporting-summary-flat.pdf

Life sciences study design

All studies must disclose on these points even when the disclosure is negative.

Sample size	No statistical methods were used to predetermine sample size. For each assay, sample size was determined based on variability across independent experiments. Sample sizes are described in the figure legends.
Data exclusions	No data were excluded.
Replication	All experiments were conducted at least two times and could be reliably reproduced.
Randomization	Animals were randomly allocated into different experimental groups based on an equal average tumor burden. During in vitro experiments, all samples were randomly allocated into experimental groups.
Blinding	Investigators collecting tumor measures were blinded to group allocation during treatment and data collection. Blinding was not performed for the in vivo tumor data analyses or the in vitro data analysis as the investigators needed to know the treatment groups in order to perform the study, and the data analyses were based on objectively measurable data.

Reporting for specific materials, systems and methods

We require information from authors about some types of materials, experimental systems and methods used in many studies. Here, indicate whether each material, system or method listed is relevant to your study. If you are not sure if a list item applies to your research, read the appropriate section before selecting a response.

Materials & experimental systems

n/a	Involved in the study
☐	☒ Antibodies
☐	☒ Eukaryotic cell lines
☒	☐ Palaeontology and archaeology
☐	☒ Animals and other organisms
☒	☐ Clinical data
☒	☐ Dual use research of concern

Methods

n/a	Involved in the study
☒	☐ ChIP-seq
☐	☒ Flow cytometry
☒	☐ MRI-based neuroimaging

Antibodies

Antibodies used

1. Chicken anti-GFP (Abcam, cat. no. ab13970)
2. Rabbit anti-desmin (Abcam, cat. no. ab15200)
3. Rat anti-CD31 (Santa Cruz, cat. no. SC18916)
4. Mouse anti-alpha-Smooth Muscle Actin (SMA) (Sigma-Aldrich, cat. no. A5228)
5. Rabbit anti-TEM8 monoclonal antibody (AbCam, clone EPNCI-R173-37, cat no. ab241067)
6. m825 human anti-TEM8 antibody (in-house, St. Croix laboratory, NCI, NIH, PMID: 29863500)
7. m830 human anti-TEM8 antibody (in-house, St. Croix laboratory, NCI, NIH, this study)
8. SB5 mouse anti-TEM8 monoclonal antibody (in-house, PMID: 14871805)
9. Anti-CMG2 antibody (clone 1H9) (Steve Leppla, NIAID, NIH)
10. Rabbit anti-GLUL antibody (clone D2O3F) (Cell Signaling Technology, cat no. 80636)
11. Anti-PEPD Antibody (clone A-3) (Santa Cruz biotechnology, cat. no. sc-390042)
12. Biotin-labelled donkey Anti-Mouse IgG (Jackson ImmunoResearch, cat. no. 715-065-151)
13. Biotin-labelled goat anti-mouse Fcg (Jackson ImmunoResearch, cat. no. 115-065-164)
14. Biotin goat anti-human (Jackson ImmunoResearch, cat. no. 109-065-088)
15. Biotin-labelled donkey anti-rabbit (Jackson Immunoresearch, cat no. 711-065-152)
16. Alexa 647 donkey anti-rat (Jackson Immunoresearch, cat. no. 112-605-175)
17. FITC goat anti-chicken (Jackson Immunoresearch, cat. no. 103-095-155)
18. FITC goat Anti-human IgG (H+L) (Jackson ImmunoResearch, cat. no. 109-095-088)
19. Alexa Fluor 488 anti-Fluorescein/Oregon Green Antibody, (ThermoFisher, cat. no. A-11096)
20. Alexa Fluor 488 donkey anti-Goat IgG (ThermoFisher, cat. no. A1105)
21. Alexa Fluor 647 anti-mouse CD31 Antibody (BioLegend, Cat. no. 102516)
22. Anti-Ki67 antibody (Abcam, ab15580)
23. Anti-beta-actin (Santa Cruz, sc-376401)
24. Alexa 350 Streptavidin (Thermo Fisher, S11249)
25. Texas red-streptavidin (Vector Laboratories, SA-5006-1)
26. Mouse anti-collagen (Southern Biotech, 1441-01)
27. Alexa 594 labeled goat anti-mouse (Jackson ImmunoResearch, 715-585-151)
28. 488-linked goat anti-FITC (ThermoFisher, A11055)
29. rabbit anti-collagen (Rockland, 600-401-103)
30. 594 labeled donkey anti-rabbit (Jackson ImmunoResearch, 711-585-152)

Validation

All commercial antibodies were verified by the supplier and each lot has been quality tested. Validation data are available on the manufacturer's website.
1. Chicken anti-GFP (https://www.abcam.com/gfp-antibody-ab13970.html)
2. Rabbit anti-desmin (https://www.abcam.com/desmin-antibody-cytoskeleton-marker-ab15200.html)
3. Rat anti-CD31 (https://www.scbt.com/p/pecam-1-antibody-mec-13-3)
4. Mouse anti-alpha-Smooth Muscle Actin (SMA) (https://www.sigmaaldrich.com/US/en/product/sigma/a5228)
5. Rabbit anti-TEM8 monoclonal antibody (https://www.abcam.com/tem8atr-antibody-epnci-r173-37-ab241067)
6. m825 human anti-TEM8 antibody (in-house, PMID: PMID: 29863500)
7. m830 human anti-TEM8 antibody (in-house, this study)
8. SB5 mouse anti-TEM8 monoclonal antibody (in-house, PMID: 14871805)
9. Anti-CMG2 antibody (clone 1H9) (PMID: 29863500)
10. Rabbit anti-GLUL antibody (clone D2O3F) (https://www.cellsignal.com/products/primary-antibodies/glul-d2o3f-rabbit-mab/80636)
11. Anti-PEPD Antibody (clone A-3) (https://www.scbt.com/p/pepd-antibody-a-3)
12. Biotin-labelled donkey Anti-Mouse IgG (https://www.jacksonimmuno.com/catalog/products/715-065-151)
13. Biotin-labelled goat anti-mouse Fcg (https://www.jacksonimmuno.com/catalog/products/115-065-164)
14. Biotin goat anti-human (https://www.jacksonimmuno.com/catalog/products/109-065-088)
15. Biotin-labelled donkey anti-rabbit (https://www.jacksonimmuno.com/catalog/products/711-065-152)
16. Alexa 647 donkey anti-rat (https://www.jacksonimmuno.com/catalog/products/112-605-175)
17. FITC goat anti-chicken (https://www.jacksonimmuno.com/catalog/products/103-095-155)
18. FITC goat Anti-human IgG (H+L) (https://www.jacksonimmuno.com/catalog/products/109-095-088)
19. Alexa Fluor 488 anti-Fluorescein/Oregon Green Antibody (https://www.thermofisher.com/antibody/product/Fluorescein-Oregon-Green-Antibody-Polyclonal/A-11096)
20. Alexa Fluor 488 donkey anti-Goat IgG (https://www.thermofisher.com/antibody/product/Goat-anti-Mouse-IgG-H-L-Cross-Adsorbed-Secondary-Antibody-Polyclonal/A-11005)

21. Alexa Fluor 647 anti-mouse CD31 Antibody (https://www.biolegend.com/en-us/products/alexa-fluor-647-anti-mouse-cd31-antibody-3094)
22. Anti-Ki67 antibody (https://www.abcam.com/ki67-antibody-ab15580.html)
23. Anti-beta-actin (https://www.scbt.com/p/beta-actin-antibody-c4?requestFrom=search)
24. Alexa 350 Streptavidin (https://www.thermofisher.com/order/catalog/product/S11249)
25. Texas red-streptavidin (https://vectorlabs.com/products/avidin/texas-red-streptavidin)
26. Mouse anti-collagen (https://www.southernbiotech.com/mouse-anti-human-type-i-collagen-unlb-4f6-1441-01)
27. Alexa 594 labeled goat anti-mouse (https://www.jacksonimmuno.com/catalog/products/715-585-151)
28. 488-linked goat anti-FITC (https://www.thermofisher.com/antibody/product/Donkey-anti-Goat-IgG-H-L-Cross-Adsorbed-Secondary-Antibody-Polyclonal/A-11055)
29. Rabbit anti-collagen (https://www.rockland.com/categories/primary-antibodies/collagen-type-i-antibody-600-401-103-0.1/)
30. 594 labeled donkey anti-rabbit (https://www.jacksonimmuno.com/catalog/products/711-585-152)

Eukaryotic cell lines

Policy information about cell lines and Sex and Gender in Research

Cell line source(s)	293, HPAC, SW620, HCT116, CT26, 4T1 and BALB/3T3 were obtained from the American Type Culture Collection (ATCC). E0771 cells were obtained from CH3 BioSystems and PC9 cells were from Sigma. B16, glioma 261 and UACC-64 (UACC) cell lines were from the DCTD Tumor Repository at NCI (Frederick, MD). MC38, RENCA, and CHO-PR230 (CHO) cell lines were gifts of Jeffrey Schlom (NCI, NIH), Jonathan M. Weiss (NCI, NIH), and Stephen H. Leppla (National Institute of Allergy and Infectious Diseases [NIAID]), respectively. TSCs were isolated as previously described (PMID: 29863500).
Authentication	Immediately upon receipt from the supplier, each cell line was expanded and immediately cryopreserved in a large master stock. Upon isolation, in-house derived TSCs were also expanded and frozen in a master stock. For each new experiment, fresh cells were revived from the master stock. Cell lines were authenticated by periodic morphology check via microscope.
Mycoplasma contamination	All cell lines were tested for mycoplasma contamination. No mycoplasma contamination was found.
Commonly misidentified lines (See ICLAC register)	No commonly misidentified cell lines are used in this study.

Animals and other research organisms

Policy information about studies involving animals; ARRIVE guidelines recommended for reporting animal research, and Sex and Gender in Research

Laboratory animals	Mice of various genetic backgrounds, ages and strains were used as detailed in the animal methods and figure legends. Both female and male TEM8 wildtype, knockout mice and conditional knockout mice were used for tumor studies. Therapeutic studies using m830 antibody were conducted using female NCr nu/nu mice. Conventional mouse housing was used with humidity from 40-50%, temperature from 68-79F and a 12h light/dark cycle. C57BL6/NCr, BALB/c, FVB-J, athymic nu/nu and C57BL6 SCID mice were used as described in the manuscript. The age of mice ranged from 3 to 12 months depending on the experiment as described in the manuscript.
Wild animals	Wild animals were not used in this study.
Reporting on sex	Both female and male TEM8 wildtype and knockout mice were used for tumor studies, and the impact of TEM8 on tumor growth suppression was observed in both females and males. Therapeutic studies using m830 antibody were conducted using female NCr nu/nu mice only because females NCr nu/nu females can be housed together after tumor randomization minimizing cage space and cage costs.
Field-collected samples	This study did not involve samples collected from the field.
Ethics oversight	All mice were bred and maintained in a pathogen free facility certified by the Association for Assessment and Accreditation of Laboratory Animal Care International, and the study was carried out in accordance with protocols approved by the NCI Animal Care and Use Committee.

Note that full information on the approval of the study protocol must also be provided in the manuscript.

Flow Cytometry

Plots

Confirm that:

☒ The axis labels state the marker and fluorochrome used (e.g. CD4-FITC).

☒ The axis scales are clearly visible. Include numbers along axes only for bottom left plot of group (a 'group' is an analysis of identical markers).

☒ All plots are contour plots with outliers or pseudocolor plots.

☒ A numerical value for number of cells or percentage (with statistics) is provided.

Methodology

Sample preparation	CHO cells, a gift of Stephen H. Leppla (National Institute of Allergy and Infectious Diseases [NIAID]), or CHO expressing wildtype or mutant TEM8 were trypsinized and labeled with m830 human IgG in PBS-BSA followed by FITC-conjugated goat anti-human IgG antibodies. Analysis was performed on a FACSCalibur Flow Cytometer (BD).
Instrument	FACSCalibur Flow Cytometer (BD)
Software	FlowJo v10.
Cell population abundance	A cell count of 10,000 events was collected for relevant cell populations after initial gating.
Gating strategy	Initial cell populations were gated for singlet and doublet cells using FSC-A/ FSC-W gating. Parental CHO or TSC-KO cells which lack target were used to set the background staining gates.

☐ Tick this box to confirm that a figure exemplifying the gating strategy is provided in the Supplementary Information.
